# Supplementary material for: Subthreshold Conduction of Disordered ZnO-Based Thin-Film Transistors
Source: Micromachines (Basel). 2023 Aug 13;14(8):1596. doi: 10.3390/mi14081596 (PMC10456918; doi:10.3390/mi14081596)
Supplement: Supplementary file 1 [file micromachines-14-01596-s001.zip › micromachines-2560007-supplementary.pdf]

Article

Subthreshold conduction of disordered ZnO-based thin-film transistors

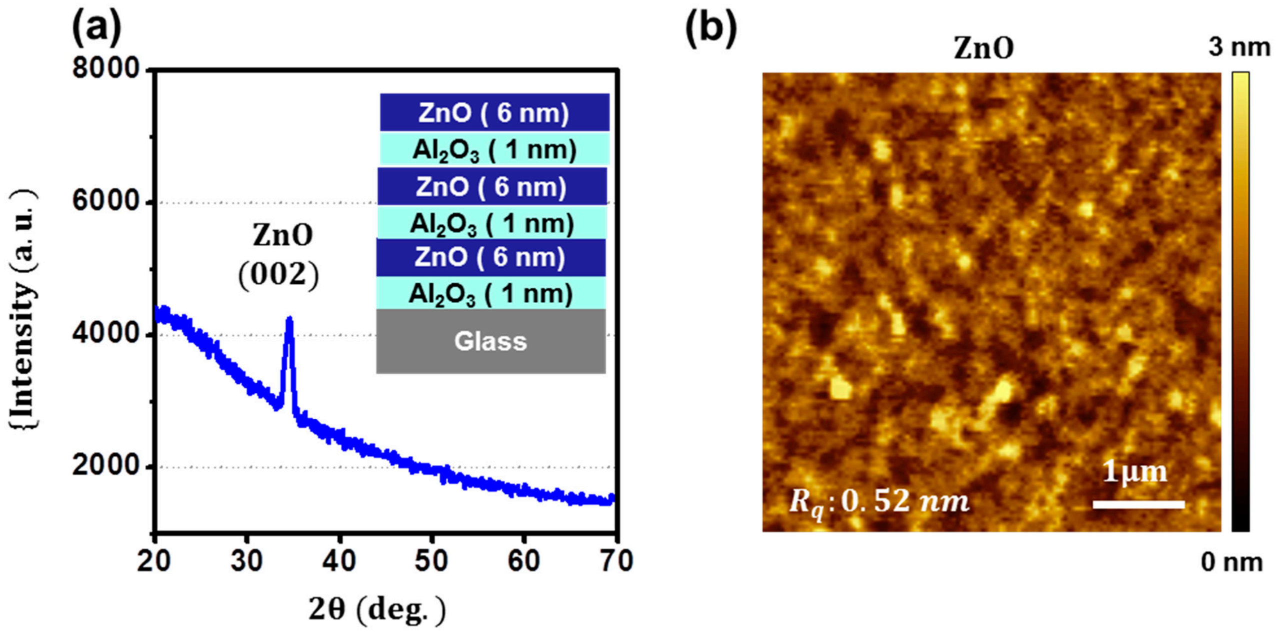

Figure S1. (a) XRD pattern of the ZnO films. (b) The surface morphology of the ZnO films.

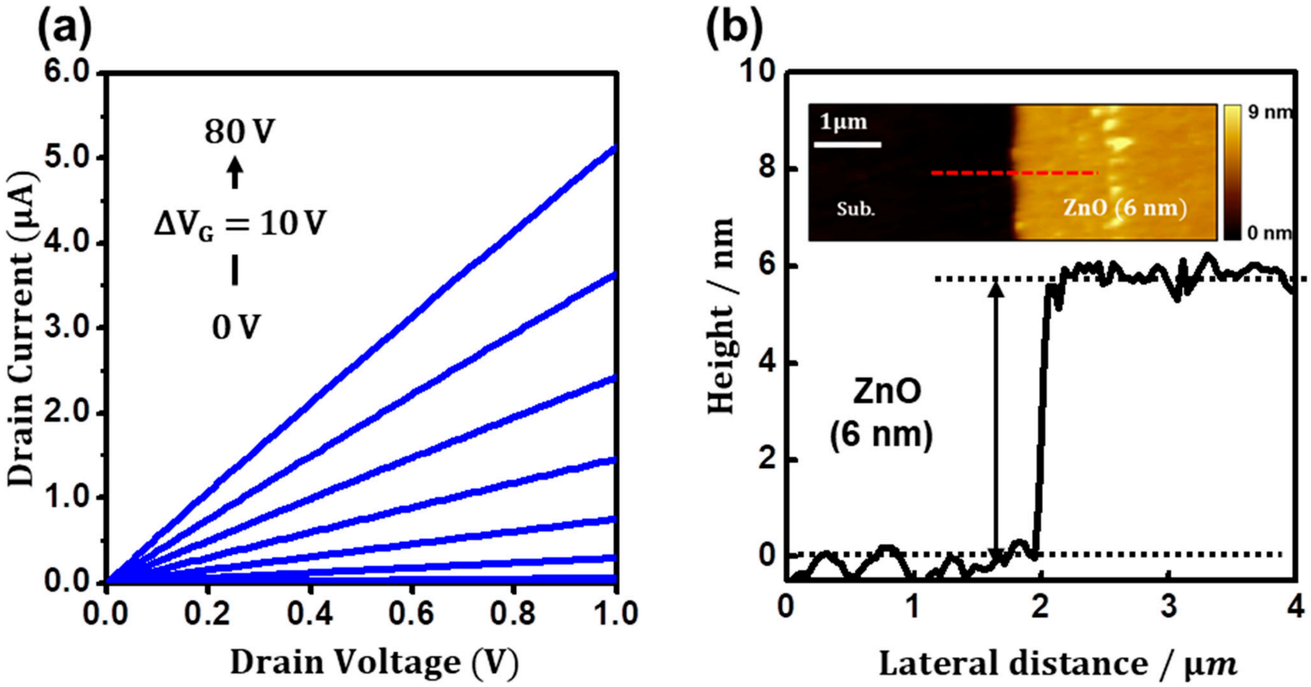

Figure S2. (a) Output characteristics of the ZnO TFTs. (b) The thickness of the ZnO films with an atomic microscope.

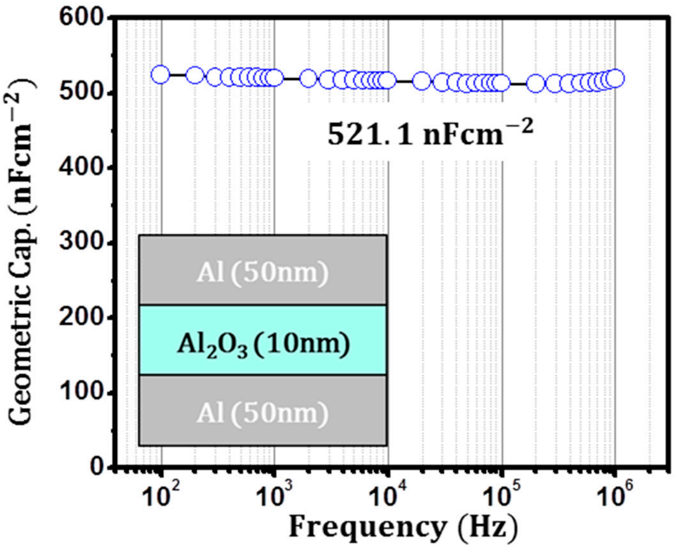

Figure S3. Geometric capacitance of the 10 nm-thin Al_2O_3 dielectric layer.
